# Ethical value conflicts in healthcare and their effects on nurses’ health, turnover intent, team effectiveness, and patient safety: a longitudinal questionnaire study

**DOI:** 10.5271/sjweh.4138

**Published:** 2024-03-01

**Authors:** Pernilla Larsman, Anders Pousette, Maria Skyvell Nilsson, Christian Gadolin, Marianne Törner, Med Sc

**Affiliations:** 1 Department of Psychology, University of Gothenburg, Gothenburg, Sweden.; 2 Department of Health Sciences, University West, Trollhättan, Sweden.; 3 School of Business, Economics and IT, University West, Trollhättan, Sweden.; 4 School of Public Health and Community Medicine, Inst. of Medicine, Sahlgrenska Academy, University of Gothenburg, Gothenburg, Sweden.

**Keywords:** ethical dilemma, moral distress, nursing, nurses’ well-being, quality of care

## Abstract

**Objective:**

Moral distress emanating from value conflicts comprising ethical dimensions pose a threat to nurses’ health and retention, as well as to the quality of care. The aim of the present study was to investigate the relationships between the frequency of ethical value conflicts (EVC), and the perceived distress when they occur, respectively, and nurses’ work-related stress, burnout symptoms, turnover intent, team effectiveness, and patient safety.

**Methods:**

A two-wave longitudinal cohort questionnaire study was performed among registered nurses at six hospitals in two Swedish regions. Cross-sectional analyses (T1) were based on 1817 nurses in 228 care units (CU), and longitudinal analyses (T1 – T2) on 965 nurses in 190 CU. Hypothesis testing was performed using multilevel controlled regression modeling.

**Results:**

The results indicated that nurses who were often exposed to EVC also to a higher extent tended to report these conflicts as stressful. Frequent exposure to EVC induced by insufficient resources, inapt organizational structures or interpersonal staff relations were cross-sectionally associated with work-related stress, burnout symptoms, turnover intent, and team effectiveness. The longitudinal analyses indicated that EVC induced by a lack of resources primarily had negative effects on nurses’ health and well-being. At the CU level, such conflicts also impaired team effectiveness. At the individual level, EVC induced by organizational constraints or interpersonal relations negatively affected care effectiveness.

**Conclusions:**

EVC are related to negative consequences in healthcare, and such processes take place both on the individual and organizational levels.

High-quality healthcare is essential for societal welfare, and a professional group central to its realization is registered nurses. The perception of moral distress, emanating from value conflicts comprising ethical dimensions (1) poses a specific threat to nurses’ health and retention, as well as to the quality of care (1–3). Moral distress is a possible outcome of exposure to ethical value conflicts (EVC). A similar concept, moral injury (4), defined as persisting distress that follows exposure to morally injurious events or morally threatening situations (5), has been found to be associated with deteriorated mental health, higher risk of burnout and psychological distress, and less well-being among healthcare workers (6, 7). Value conflicts stem from the necessity to accommodate the tension between paradoxical demands that are contradictory yet interrelated and that persist over time (8). EVC that appear at the operational level in the organization (9, 10) often present stressful goal conflicts for the employees (11). Distress due to EVC is common in healthcare organizations, and nurses have been found to perceive higher levels than other professionals (3). EVC threaten the quality of care since nurses who often face such conflicts have been found to be more emotionally exhausted, more inclined to leave their employment, more emotionally distanced from the patients, and have a more cynical attitude toward them (2).

In summary, research indicates moral distress to be associated with adverse outcomes, both in terms of nurses’ health and wellbeing, and in terms of turnover, care quality and care effectiveness (1–3, 6, 7). However, further investigation of these associations using longitudinal study designs is warranted. In-depth knowledge regarding the effects of EVC and moral distress on the actual quality of care is needed (1). There is a lack of knowledge regarding where and when the EVC occur, and further research is needed investigating the relationship between various EVC and their associated strain (12). Better understanding of the types of EVC that are common in nursing work, and their effects on nurses’ health, as well as on care effectiveness and quality, is important. Such knowledge allows the development of strategies to minimize the occurrence of EVC and to support the nurses’ ability to resolve such situations in a manner that is satisfactory to their health and well-being, as well as to the care quality and effectiveness. Thus, the aim of the present study was to investigate the relationships between the frequency of EVC and, the perceived distress when they occur, respectively, and nurses’ work-related stress, burnout symptoms, turnover intent, team effectiveness, and patient safety.

## Methods

A two-wave pen-and-paper questionnaire survey was directed to all registered nurses within six hospitals with secondary and tertiary care in two regions in Sweden. Both in- and outpatient care was included, with care units (CU) within a wide range of medical specialities such as medicine; surgery; orthopedics; and emergency, pediatric, intensive, and infectious disease care. The first wave of data was collected during November 2019 – January 2020 (T1) and the second one year later (T2). The response rates were 54% at T1 and 48% at T2. In total, 1030 nurses completed the questionnaire on both occasions. Of these, 965 nurses had remained working at the same CU on both occasions (ie, 94% of the original longitudinal study sample).

### Participants

The cross-sectional analyses were based on data from 1817 nurses (86% females) at 228 CU (each containing 1–47 respondents) responding at T1. The nurses’ median age was 42 [mean 42.4, standard deviation (SD) 12.2] years. About half (54%) had been employed by their present regional healthcare organization for >10 years. On average, they had been working as nurses for 15 years (mean 15.1, SD 11.4 years); 62% had been working as nurses for ≥10 years.

The longitudinal analyses were based on data from 965 nurses (85% females) within 190 CU (each containing 1–31 respondents) who had remained working at the same CU at both measurement occasions. About 25% of these respondents reported having worked within another CU for some time during the measurement period because of the then ongoing COVID-19 pandemic. The nurses’ median age was 45 (mean 44.3, SD 12.0) years. More than half of them (61%) had been employed by their present regional healthcare organization for >10 years. On average, they had been working as nurses for 17 (mean 16.8, SD 11.4) years; 69% had been working as nurses for ≥10 years.

### Measures

EVC were investigated using a combination of items from the Moral Distress Scale (MDS-R) (13, 14) and items that were constructed within the present project (15), based on the results from Kävlemark et al (16). The purpose was, as completely as possible, to capture the types of EVC occurring in nurses’ work. An expert panel of eight healthcare professionals in different positions, professions, and medical specialties within Swedish healthcare reviewed and completed the total set of items. This panel testing resulted in a scale consisting of 19 items; 8 were from the MDS-R (13, 14), but some were slightly modified to fit a Swedish context. The entire questionnaire (also including the variables below) was further tested in a pilot study among 385 nurses at 34 hospital CU in four Swedish regions that did not participate in the full-scale study. The questionnaire items showed good psychometric properties, and only minor revisions were implemented.

All 19 items present respondents with a number of different situations potentially comprising EVC and ask them to rate how often they had experienced each such situation (frequency) as well as how stressful they perceived each such situation to be when it occurred (distress). All items had five fixed response alternatives for frequency (range 0–4) from “never” to “very often” and for distress from “not at all stressful” to “highly stressful”. Exploratory factor analyses indicated that 17 of these items could be modelled as two separate factors (15): *EVC frequency resources* comprising EVC induced by insufficient resources, with sample item *“*be forced to prioritize between patients due to too high patient occupancy in the hospital”) (9 items: in the cross-sectional sample α=0.89 at T1, and in the longitudinal sample α=0.89 at both T1 and T2); and *EVC frequency structures* comprising EVC induced by inapt organizational structures or interpersonal staff relations, with sample item *“*ignore situations in which patients have not been given adequate information to ensure informed consent”) (8 items: in the cross-sectional sample α=0.82 at T1 and in the longitudinal sample α=0.82 at T1 and 0.81 at T2). The variable *EVC distress* consisted of distress ratings of all 17 items (in the cross-sectional sample α=0.94 at T1, and in the longitudinal sample α=0.95 at both T1 and T2). These mean level index variables were calculated such that high values indicate a high frequency of EVC situations and high perceived distress.

Perceived *work-related stress* was measured using the two-dimensional mood adjective checklist (17, 18), consisting of the six items “rested”, “relaxed”, “calm”, “tense”, “stressed”, and “pressured” (in the cross-sectional sample α=0.93 at T1, and in the longitudinal sample α=0.93 at both T1 and T2). The respondents were instructed to think about how they usually feel at the end of a normal workday. There were six fixed response alternatives (range 0–5) from ”not at all” (0) to “to a very high extent” (5). Items were coded such that high values indicate high levels of stress.

Self-reported *burnout symptoms* (physical and emotional exhaustion) were assessed using four items (in the cross-sectional sample α=0.89 at T1, and in the longitudinal sample α=0.89 at T1 and 0.90 at T2) from the Copenhagen psychosocial questionnaire (COPSOQ-II) (19) with sample items “how often have you been physically exhausted?” and “how often have you been emotionally exhausted?”. There were five fixed response alternatives (range 1–5) from “not at all” to “all of the time”. Items were coded such that high values indicate a high level of self-reported burnout symptoms.

*Turnover intent,* ie, the intention to leave the current employment, was assessed using the three items “often think about leaving my current employment”, “actively search for another job as a nurse”, and “will leave my current employment and search for another job as a nurse as soon as possible” (20, 21) (in the cross-sectional sample α=0.89 at T1, and in the longitudinal sample α=0.85 at T1 and 0.88 at T2). There were five fixed response alternatives ranging from “completely disagree” to “completely agree”.

Self-reported CU *team effectiveness* was assessed using five items (in the cross-sectional sample α=0.89 at T1, and in the longitudinal sample α=0.89 at both T1 and T2) from Gibson et al (22), slightly modified to suit the context of nursing, with sample item “My team meets the patients’ needs”. There were seven fixed response alternatives ranging from “very inaccurate” to “very accurate”.

Self-reported CU *patient safety* was assessed using a single item rating “the patient safety at your care unit” (23), with five fixed response alternatives ranging from “poor” to “excellent”.

### Statistical analyses

Bivariate correlations in cross-sectional data (T1) were calculated between the three variables representing EVC (frequency resources; frequency structures; distress), and the five outcome variables (work-related stress, burnout symptoms, turnover intent, team effectiveness, and patient safety).

Multilevel modelling was performed to investigate the prospective effects of the nurses’ perceptions of EVC aggregated to the CU, as well as the individual nurses’ deviations from those aggregate perceptions and their hypothesized outcomes, while controlling for the effects of these outcomes at baseline (T1). In this strategy, multilevel, controlled regression models with predictors at the individual and the aggregated level (CU) were tested. Separate analyses were performed for each of the five outcomes. Data for each predictor was divided into two components. The first component was the aggregated value for all respondents at the CU (between), and the second component was the difference between each respondent’s value and the CU mean (within). In the regression models the outcome was predicted by both components of the predictor, making a total of six predictors (T1 EVC frequency resources_within CU; T1 EVC frequency structures_within CU; T1 EVC distress_within CU, T1 EVC frequency resources_between CU; T1 EVC frequency structures_between CU; T1 EVC distress_between CU). A random effect within between model was included.

The multilevel models investigate prospective relationships between EVC and proposed outcomes using longitudinal data. When drawing conclusions based on longitudinal data, however, we need to ensure that the same thing is being measured in the same way over time. Therefore, (metric) factorial invariance (FI) over the two measurement occasions was considered a prerequisite for hypothesis testing. Three models were tested: one including EVC frequency (two latent factors), one including EVC distress (one latent factor), and one including the proposed multiple-item outcome variables (work-related stress, burnout symptoms, turnover intent, and team effectiveness, four latent factors). For all these models, the first manifest indicator within each latent variable was fixed to 1 to set the scales of the latent variables. All latent variables were allowed to co-vary, and error terms were allowed to auto-correlate over time. To investigate invariance, two nested models were fit and compared. Firstly, a baseline (configural invariance) model was estimated, only requiring the number and pattern of factor loadings to be equal over time. Thereafter, a metric factorial invariance (FI) model with additional equality constraints on factor loadings over time was estimated. The results of these FI tests are presented in table 1. In accordance with previous research (24) FI was evaluated investigating differences in the comparative fit index (CFI) for these consecutive models. Since adding equality constraints on factor loadings did not cause substantially worse model fit (ie, Δ CFI was <0.005 for all three models, see table 1), we concluded that no serious violations of metric invariance were indicated, ie, factor loadings did not substantially differ over time.

**Table 1:** Fit indices for tests of factorial invariance (FI) for the latent constructs investigated in the present study. [CFI=comparative fit index; RMSEA=root mean square error of approximation; SRMR=standardized root mean square residual.]

		χ^2^	CFI	RMSEA (90% CI)	SRMR	ΔCFI
Ethical value conflict frequency						
	Configural FI	χ^2^=1472.73, df = 504, P<0.001	0.941	0.045 (0.042–0.047)	0.034	
	Metric FI	χ^2^=1502.22, df = 519, P<0.001	0.940	0.044 (0.042–0.047)	0.036	0.001
Ethical value conflict intensity						
	Configural FI	χ^2^=2226.70, df = 509, P<0.001	0.910	0.059 (0.057–0.062)	0.040	
	Metric FI	χ^2^=2252.55, df = 525, P<0.001	0.910	0.058 (0.056–0.061)	0.041	0.000
Stress, burnout, turnover intent, team effectiveness						
	Configural FI	χ^2^=1911.93, df = 548, P<0.001	0.950	0.051 (0.048–0.053)	0.047	
	Metric FI	χ^2^=1956.80, df = 562, P<0.001	0.948	0.051 (0.048–0.053)	0.050	0.002

Invariance testing was performed using Mplus v.8.4 employing the (full information) maximum likelihood estimator. All other statistical analyses were performed in SPSS version 28, IBM Corp, Armonk, NY, USA.

## Results

### Frequency of and distress due to ethical value conflicts

Table 2 shows how often the 19 different EVC occurred. The three most frequent root causes were (i) lack of provider continuity (mean 1.98); (ii) unsafe levels of care provider staffing (mean 1.94); and (iii) patients having to wait (mean 1.85). As can be seen in table 2, situations referring to EVC frequency resources were more frequent than situations referring to EVC frequency structures. Most value conflicts occurred at the group level at least sometimes. Almost all nurses (99%) reported the occasional occurrence of at least one of the EVC. More than one third of the nurses (37%) reported that at least one such conflict occurred very often.

**Table 2:** Root causes of ethical value conflict (EVC) frequency (f) and distress (d). Factor 1 comprises EVC induced by insufficient resources (EVC frequency resources) while factor 2 comprises EVC induced by inapt organizational structures or interpersonal staff relations (EVC frequency structures). [NA=not available; ICC=intraclass correlation; SD=standard deviation.]

Factor		Frequency					Distress				R (f,d)
		Rank	Mean	SD	ICC		Rank	Mean	SD	ICC	
NA	lack of provider continuity	1	1.98	1.17	0.19		3	2.93	1.08	0.05	0.42
1	unsafe levels of care provider staffing	2	1.94	1.22	0.27		1	3.15	1.08	0.14	0.48
1	patients having to wait	3	1.85	1.15	0.21		13	2.48	1.17	0.09	0.41
1	forced to do administrative work	4	1.83	1.34	0.24		7	2.59	1.26	0.13	0.56
2	incompetent healthcare providers	5	1.66	1.16	0.22		4	2.92	1.15	0.07	0.41
1	lack of resources	6	1.62	1.13	0.22		2	2.95	1.13	0.10	0.47
1	prioritize between patients	7	1.52	1.37	0.38		12	2.51	1.37	0.18	0.49
1	refrain from conversations with patients and their family	8	1.47	1.22	0.30		9	2.55	1.27	0.16	0.47
2	follow physicians’ prescriptions that do not benefit the patient	9	1.21	1.09	0.26		11	2.51	1.34	0.10	0.45
1	not feel confident that care is of high quality	10	1.16	1.02	0.19		5	2.64	1.30	0.10	0.41
2	violating formal rules and regulations	11	1.15	1.01	0.07		19	1.71	1.24	0.03	0.22
1	feel unqualified	12	1.14	1.11	0.29		6	2.59	1.41	0.10	0.48
2	formal rules and routines as barriers	13	1.00	0.98	0.08		18	2.13	1.34	0.03	0.43
2	follow the family’s wishes that do not benefit the patient	14	0.99	0.93	0.18		16	2.38	1.36	0.08	0.38
NA	disregard the patients’ integrity	15	0.93	1.02	0.19		15	2.39	1.37	0.11	0.32
2	witness healthcare providers giving “false hope”	16	0.93	0.88	0.11		10	2.51	1.28	0.03	0.23
1	provide care that does not correspond to that of a professional nurse	17	0.86	0.98	0.14		8	2.56	1.42	0.10	0.34
2	ignore situations in which patients have not been given adequate information	18	0.83	0.88	0.10		17	2.32	1.30	0.06	0.27
2	avoid taking action when a medical error has not been reported	19	0.65	0.76	0.01		14	2.45	1.31	0.03	0.17

Table 2 also shows how stressful these different EVC were perceived when they did occur. The three root causes inducing the highest distress were (i) unsafe levels of care provider staffing (mean 3.15); (ii) lack of resources (mean 2.95); and (iii) lack of provider continuity (mean 2.93). Since the response scale varied from 0 (not at all stressful) to 4 (highly stressful), most value conflicts were generally perceived as rather stressful when they did occur.

As can be seen in table 2, the rank orders of EVC frequency and distress overlap to some extent. This indicates that the EVC which occurred often also tended to be perceived as more stressful when they did occur. There were, however, some notable exceptions to this, eg, “patients having to wait” occurred relatively often, but when it did occur it was perceived as less stressful. “Provide care that does not correspond to that of a professional nurse” was a relatively rare situation. However, when it did occur it was perceived as rather stressful.

There were associations between EVC frequency and distress (see table 1). These associations ranged from small (*r*=0.17) to moderate (*r*=0.56) for the different types of EVC. This indicates that nurses who often were exposed to EVC also tended to report these conflicts as more stressful.

Intraclass correlations (ICC) are reported in table 2, and were 0.01–0.38 for EVC frequency, with ICC generally being higher for EVC frequency resources. ICC for EVC distress were 0.03–0.18.

### Outcomes of ethical value conflicts: correlation analyses

The results from cross-sectional correlation analyses are reported in table 3. All aspects of EVC (ie, frequency resources, frequency structures, and distress) were cross-sectionally associated with work-related stress, self-reported burnout symptoms, turnover intent, and CU team effectiveness, such that nurses who perceived a high frequency of EVC, and/or a high distress in relation to such conflicts, also, in general, perceived higher work-related stress, more burnout-symptoms, higher intention to leave the employment, and lower CU team effectiveness. EVC frequency was cross-sectionally associated with patient safety, ie, nurses who perceived a high frequency of EVC also, in general, rated the patient safety at their CU as lower. There was no cross-sectional association between EVC distress and patient safety.

**Table 3:** Correlations between ethical value conflicts (EVC) and work-related stress, burnout symptoms, turnover intent, care-unit team effectiveness, and patient safety.

	1.	2.	3.	4.	5.	6.	7.
1. EVC frequency resources							
2. EVC frequency structures	0.71**						
3. EVC distress	0.39**	0.32**					
4. Stress	0.52**	0.33**	0.29**				
5. Burnout	0.52**	0.38**	0.26**	0.66**			
6. Turnover intent	0.34**	0.29**	0.12**	0.38**	0.43**		
7. Team effectiveness	-0.42**	-0.36**	-0.12**	-0.31**	-0.28**	-0.29**	
8. Patient safety	-0.24**	-0.32**	-0.00	-0.21**	-0.20**	-0.32**	0.40**

** P<0.01

### Outcomes of ethical value conflicts: multilevel, controlled regression models with predictors at the individual and the aggregated level

The results from the longitudinal multilevel controlled regression models with predictors at the individual level and aggregated level are reported in table 4. These analyses indicate EVC distress aggregated to the CU to be prospectively related to work-related stress, ie, nurses working in CU where the EVC distress was rated as high at T1, perceived a higher work-related stress at T2. Nurses who reported higher levels of EVC frequency resources at T1 *relative to other nurses in their* CU reported higher levels of burnout symptoms at T2. As regards CU team effectiveness, EVC frequency structures had a negative prospective effect within CU, while EVC frequency resources had a negative prospective effect between CU. This indicates that nurses who reported less EVC frequency structures, relative to other nurses within their CU at T1, rated the team effectiveness as higher at T2, while nurses working in CU with less EVC frequency resources at T1 rated the team effectiveness as higher at T2.

**Table 4:** Longitudinal multilevel regression analyses (one model per outcome), random effect within between models (REWB), with predictors at the individual level and at the aggregate level. [EVC=ethical value conflicts; freq=frequency; SE=standard error; T=time]

	Stress				Burnout				Turnover intent				Effectiveness				Patient safety		
	Estimate	SE	P-value		Estimate	SE	P-value		Estimate	SE	P-value		Estimate	SE	P-value		Estimate	SE	P-value
Within-level predictors																			
T1 Autoregressive	0.66	0.03	<0.001		0.66	0.03	<0.001		0.69	0.03	<0.001		0.49	0.03	<0.001		0.57	0.03	<0.001
T1 EVC freq resources	0.05	0.05	0.31		0.16	0.05	<0.01		0.05	0.06	0.40		0.01	0.05	0.90		0.02	0.03	0.62
T1 EVC freq structures	0.09	0.06	0.13		-0.08	0.06	0.16		0.03	0.07	0.71		-0.13	0.05	0.01		-0.07	0.04	0.08
T1 EVC distress	0.03	0.03	0.36		0.02	0.03	0.49		-0.01	0.03	0.69		0.04	0.03	0.16		0.01	0.02	0.68
Between-level predictors																			
T1 EVC freq resources	0.13	0.08	0.11		0.13	0.08	0.11		0.07	0.10	0.52		-0.19	0.07	0.01		0.02	0.06	0.72
T1 EVC freq structures	-0.10	0.11	0.36		-0.13	0.11	0.25		0.07	0.14	0.60		-0.08	0.10	0.41		-0.13	0.08	0.12
T1 EVC distress	0.14	0.07	0.05		0.08	0.07	0.25		-0.07	0.09	0.39		0.12	0.06	0.06		0.01	0.05	0.89
Random parameters																			
Residual (within-level)	0.46	0.02	<0.001		0.42	0.02	<0.001		0.61	0.03	<0.001		0.38	0.02	<0.001		0.20	0.01	<0.001
Intercept (between-level)	0.02	0.01	0.05		0.03	0.01	0.01		0.05	0.02	0.01		0.02	0.01	0.09		0.02	0.01	0.001
Loglikelihood	1968.55				1889.89				2260.65				1796.26				1215.91		

No effects of EVC on turnover intent or on patient safety were found in these analyses.

## Discussion

Consistent with previous research (6, 25, 26), the present study indicates ethically challenging situations to be common in nursing. Although almost all nurses experienced EVC, each specific type of conflict investigated in the preset study was not commonly occurring for all nurses. When EVC did occur, they were perceived as stressful. This is consistent with previous studies (2, 27). It has been suggested that the distress persists also after the stress inducing situation has passed, creating cumulative moral distress (2). This is in accordance with our results, indicating that nurses who often were exposed to EVC also tended to report these conflicts as more stressful. Moral distress thus tends to persist and accumulate as new EVC occur, and nurses who often are exposed to EVC become increasingly distressed by them due to the cumulated load.

Exploratory factor analyses indicate that the EVC included in the present study can be modelled as two distinct factors: EVC induced by insufficient resources and EVC induced by inapt organizational structures or interpersonal staff relations (15). These results are similar to those found in a recent literature review, demonstrating that nurses experience EVC associated with the nurse–patient relationship, organizational structures, and with collaboration with colleagues (26). ICC calculated in the present study were, in general, higher for EVC frequency resources, indicating that particularly EVC induced by insufficient resources can be linked to processes taking place at an organizational level.

The two different types of EVC seem to be related to somewhat different outcomes. *EVC induced by insufficient resources* were cross-sectionally associated with work-related stress and burnout symptoms, and thus seem to be primarily related to nurses’ health and well-being. EVC distress aggregated to the CU level showed a prospective negative effect on work-related stress, ie, a shared perception of a high level of EVC distress at the CU was associated with increased work-related stress over time. EVC induced by insufficient resources also showed a prospective negative effect on burnout symptoms. These results are in line with previous research highlighting insufficient resources as a source of ethical conflict (6, 28, 29) and indicating moral distress/moral injuries to be associated with burnout (1, 2, 7, 30, 31). In addition to this, in CU with more EVC induced by insufficient resources at T1, the CU team effectiveness was rated as lower at T2.

*EVC induced by inapt organizational structures or interpersonal staff relations* appear to be primarily related to healthcare effectiveness and quality. Those types of EVC, as well as the resulting distress, were cross-sectionally associated with CU team effectiveness, whereas only EVC frequency was associated with patient safety. However, previous research indicates moral distress also to be negatively associated with patient safety (32). EVC induced by inapt organizational structures or interpersonal staff relations were also prospectively negatively related to CU team effectiveness.

A possible explanation for EVC frequency resources being primarily related to nurses’ health and well-being, and EVC frequency structures primarily related to healthcare effectiveness and quality, is that nurses to some extent buffer the effects of insufficient resources on care efficiency and quality. They do this by running faster and working harder, which in the long run has detrimental effects, such as burnout. It is probably not to the same extent possible for nurses to mitigate the effects of inapt organizational structures, and such inadequacies will therefore have a more direct negative impact on CU effectiveness and patient safety. This is in line with previous research (33) suggesting that the detrimental impact on CU effectiveness and patient care of ill-suited organizational structures is more difficult for nurses to counteract compared to the effects of insufficient resources.

The overall findings of the present study indicate a significant need for healthcare organizations to create conditions that can reduce EVC among nursing staff. The recurring experience of EVC accumulates over time and impacts nurses’ health and well-being as well as care efficiency. To mitigate these effects, healthcare organizations should ensure proper resource allocation, aiming to protect the health and well-being of their nurses. Further, fostering healthy organizational conditions and interpersonal relationships can positively impact care effectiveness. Focus group interviews conducted among a subsample of nurses in the CU included in the present study highlighted perceived organizational support as a critical condition for improving nurses’ ability to resolve EVC (15). These results highlight the need to address factors on the organizational level to support nurses’ ability to handle and resolve EVC among nurses in a manner that does not induce moral distress.

### Study strengths and limitations

An important strength of the present study is its longitudinal design, which allows us to investigate prospective relationships between EVC and potential outcomes, while controlling for autoregressive effects. Furthermore, the multilevel analysis strategy employed allows us to investigate and disentangle prospective effects on both individual and group (CU) levels. The analyses are based on a fairly large sample, with acceptable response and attrition rates.

All data included in the present analyses were obtained from a single source using a single method of data collection (ie, all data consisted of self-reported questionnaire data), which may lead to common-method variance, potentially inflating the associations between the study variables, in particular in the cross-sectional analyses.

The present study focuses on nurses within two regions in Sweden. Furthermore, the sample largely consists of female nurses with high seniority. This poses limits to the representativeness of our sample, and how these results can generalize to other populations and contexts. Although this must be acknowledged as a limitation of the present study, it seems less severe given that the results of previous studies both within a Swedish (27, 34) and other (3, 6, 7) contexts, as well as recent literature reviews (12, 26) outline similar types of value conflicts as those investigated here.

Follow-up (T2) measurements were collected during one of the peaks of the COVID-19 pandemic (35). Previous research indicates that moral injury and distress among healthcare staff increased during the pandemic (4). A mixed methods study among CU managers in the hospitals included in the present research project shows that this pandemic entailed a clear focus on patient care that unified the staff around what was perceived as the core of healthcare work, and solidarity and social responsibility within work groups (35). Thus, in examining the outcomes of exposure to EVC and moral distress, the pandemic may have had both exacerbating and effect-masking consequences. For example, while both EVC frequency and distress were cross-sectionally associated with nurses’ turnover intent, there were no such prospective effects. These results are contrary to previous research indicating turnover intent and actual turnover to be associated with moral distress (2, 3). Our lack of such results may be due to (temporary) solidaric suspension of any intentions to leave the employment during an ongoing pandemic, when all healthcare workers were badly needed.

The present study focuses on the intention to leave the current employment. It is possible that exposure to EVC is related to an intention to leave the nursing profession entirely. This is a topic for future research, focusing on the potential impact of moral distress on the nursing and, indeed, the entire sector. Future studies may also benefit from using longitudinal designs with more and more frequent measures (eg, including daily ratings of moral distress and well-being) to further understand the mechanisms and the time lags involved in the development of moral distress and associated outcomes.

## Data Availability

All data analyzed during this study are available from the corresponding author upon reasonable request.
